# HIV-1 capsid uncoating initiates after the first strand transfer of reverse transcription

**DOI:** 10.1186/s12977-016-0292-7

**Published:** 2016-08-22

**Authors:** Ophélie Cosnefroy, Philip J. Murray, Kate N. Bishop

**Affiliations:** 1 Retroviral Replication Laboratory, The Francis Crick Institute, Mill Hill Laboratory, The Ridgeway, Mill Hill, London, NW7 1AA UK; 2 Division of Mathematics, University of Dundee, Dundee, DD1 4HN UK

**Keywords:** HIV-1, Capsid, Uncoating, Reverse transcription, Mathematical modelling

## Abstract

**Background:**

Correct disassembly of the HIV-1 capsid shell, called uncoating, is increasingly recognised as central for multiple steps during retroviral replication. However, the timing, localisation and mechanism of uncoating are poorly understood and progress in this area is hampered by difficulties in measuring the process. Previous work suggested that uncoating occurs soon after entry of the viral core into the cell, but recent studies report later uncoating, at or in the nucleus. Furthermore, inhibiting reverse transcription delays uncoating, linking these processes.

**Results:**

Here, we have used a combined approach of experimental interrogation of viral mutants and mathematical modelling to investigate the timing of uncoating with respect to reverse transcription. By developing a minimal, testable, model and employing multiple uncoating assays to overcome the disadvantages of each single assay, we find that uncoating is not concomitant with the initiation of reverse transcription. Instead, uncoating appears to be triggered once reverse transcription reaches a certain stage, namely shortly after first strand transfer.

**Conclusions:**

Using multiple approaches, we have identified a point during reverse transcription that induces uncoating of the HIV-1 CA shell. We propose that uncoating initiates after the first strand transfer of reverse transcription.

**Electronic supplementary material:**

The online version of this article (doi:10.1186/s12977-016-0292-7) contains supplementary material, which is available to authorized users.

## Background

Following maturation and cleavage of the HIV-1 Gag polyprotein, capsid (CA) forms a highly ordered hexameric lattice that generates a fullerene cone encasing the viral genome and associated proteins, known as the viral core [1–3]. This is released into the cytoplasm upon HIV-1 infection and the viral RNA is reverse transcribed by HIV-1 reverse transcriptase (RT) into double stranded DNA that is integrated into the host cell genome. As the outer face of the core, CA is likely involved in trafficking the core to the nucleus [4] and protecting the viral nucleic acid from cytosolic sensors [5, 6]. However, the CA lattice can be recognised by cellular restriction factors that inhibit replication [7]. An intact viral core is thought too large to enter the nucleus via a nuclear pore and thus, shedding of the CA shell is an essential part of the retroviral life cycle, and is termed uncoating [8]. The mechanism of uncoating, when and where in the cell it occurs, and which, if any, host proteins contribute to the process are still largely unknown.

Originally, uncoating was believed to be a passive process that started once the viral envelope was removed. More recently, inhibiting reverse transcription was reported to delay uncoating [9, 10]. Microtubule-facilitated uncoating has also been proposed [11, 12]. Other groups have suggested that the core must reach the nucleus to uncoat [13, 14], implying that uncoating follows reverse transcription. Regardless of timing, these findings concur that uncoating is a regulated process requiring a trigger. Indeed, perturbing uncoating is detrimental to viral infectivity [5, 15, 16] and therefore may represent a novel therapeutic target. Identifying what initiates uncoating is critical to understanding the role of CA and uncoating in HIV-1 replication.

HIV-1 replication can be followed by measuring the progress of reverse transcription. Reverse transcription is a complex process, requiring the polymerase and RNase H enzymatic activities of RT along with two strand transfer events [17]. Here, we set out to investigate how uncoating is linked to reverse transcription. As there is no definitive assay to measure uncoating, we used a variety of techniques, including all existing uncoating assays and mathematical modelling. We established a simple, kinetic model of reverse transcription and uncoating based on a minimal set of assumptions. This enabled us to formulate and test alternative hypotheses, monitor consistency between data sets and make predictions that confirmed our experimental studies using HIV-1 mutants. Ultimately, we propose that uncoating is triggered following the first strand transfer of reverse transcription.

## Results

### Nevirapine treatment inhibits uncoating

**Fig. 1:**
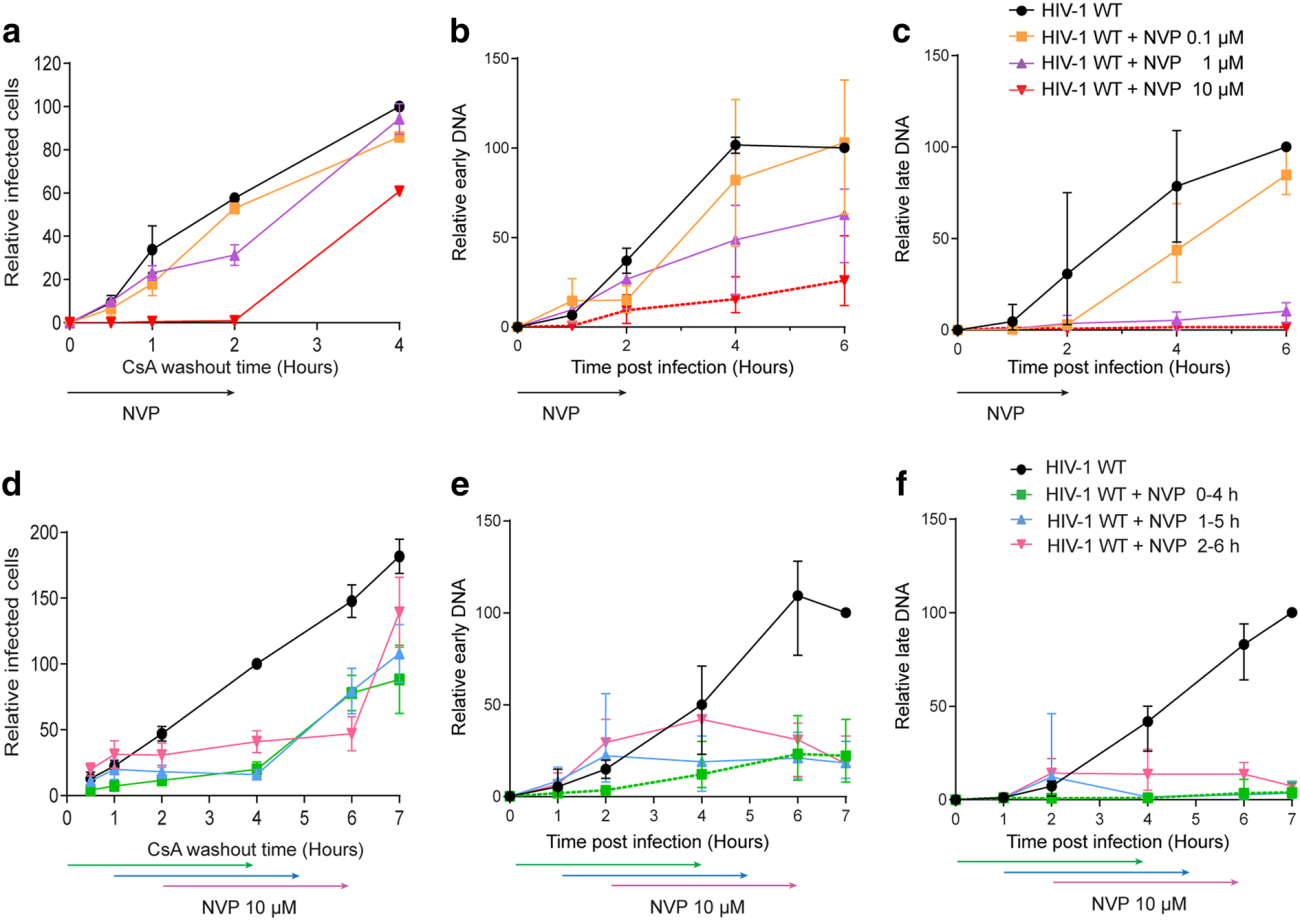
Effect of NVP on cDNA synthesis and uncoating. **a** CsA washout assay. OMK cells were infected with GFP-HIV VLP in the presence of CsA and increasing concentrations of NVP. CsA was removed from each sample at the indicated time and NVP was removed from all samples 2 h.p.i. After 72 h, the percentage of GFP positive cells was measured for each sample and plotted relative to the percentage of GFP positive cells following infection in the absence of NVP when CsA was removed 4 h.p.i. **b**, **c** 293T cells were infected with GFP-HIV VLP in the presence of increasing concentrations of NVP. NVP was removed from the media after 2 h. At the indicated times post-infection, the level of cDNA corresponding to **a** early (−sscDNA) or **b** late ((+)strand) reverse transcription products were analysed by qPCR. **d** CsA washout assay as in (**a**), except that 10 μM NVP was added for 4 h starting at 0, 1 or 2 h.p.i. The percentage of GFP positive cells for each sample was plotted relative to the percentage of GFP positive cells following infection in the absence of NVP when CsA was removed 7 h.p.i. **e**, **f** 293T cells were infected with GFP-HIV VLP in the presence of 10 μM NVP for 4 h starting at 0, 1 or 2 hp.i. Early (**e**) and late (**f**) cDNA was analysed by qPCR. *Each panel* shows the mean and SEM of at least 3 independent experiments

To confirm that uncoating is linked to reverse transcription, we performed experiments to perturb reverse transcription and measure uncoating. To do this, we utilised the non-nucleoside RT inhibitor nevirapine (NVP) to modulate reverse transcription, and took advantage of the ability of TRIMCyp to bind the HIV-1 capsid shell and inhibit replication [18] to indirectly measure uncoating using a previously published cyclosporine A (CsA) washout assay [9]. HIV-1 particles are only sensitive to TRIMCyp inhibition until they have sufficiently uncoated [9, 18–21]. Furthermore, introducing CsA into cells blocks the interaction between TRIMCyp and CA, thus allowing replication to proceed in the presence of TRIMCyp. This can be used to measure uncoating by infecting OMK cells that contain endogenous TRIMCyp in the presence of CsA, and then measuring the ability of TRIMCyp to recognise the CA shell and restrict infection at various times post-infection by removing the CsA [9]. Figure 1a shows the effect on TRIMCyp restriction of adding different concentrations of NVP for the first two hours of infection. We observed that: (1) more cells were infected the later CsA was removed from the media presumably as more particles uncoat with time, as previously reported [9] and (2) NVP treatment increased the length of time that virus particles were sensitive to TRIMCyp in a dose-dependent manner. Further experiments were then performed varying the time of addition of 10 μM NVP (Fig. 1d). NVP was added for 4 h starting at 0, 1 or 2 h post-infection. This additionally showed that (3) delayed NVP treatment appeared to temporarily pause uncoating until NVP was removed and (4) recovery after NVP washout was incomplete. Viral cDNA was isolated from parallel infections following the same NVP treatments and quantified using qPCR (see Table 1 for products measured and primers used). This showed that synthesis of both early (−sscDNA) and late ((+)strand cDNA) were affected by the presence of NVP, as expected (Fig. 1b, c, e, f). There was a dose-dependent decrease in the amount of cDNA products produced, with a larger effect on late cDNA production (Fig. 1b, c). Delayed NVP addition also showed that the later the drug was added, the more cDNA synthesis occurred (Fig. 1e, f), although the block to reverse transcription continued even after NVP was removed from the media. Interestingly, the sensitivity to TRIMCyp in the CsA washout assay reflected the levels of viral cDNA suggesting that the two events were linked. However, from these analyses, it is difficult to assess which aspect of reverse transcription leads to uncoating. For example, uncoating may be triggered by the initiation of reverse transcription or by the completion of cDNA synthesis. Therefore, to address whether uncoating is concurrent with reverse transcription or if it begins once reverse transcription has reached a certain point, we measured the effect on uncoating of blocking reverse transcription at various times.

**Table 1:** Real-time PCR primers and probes

Early: Strong stop cDNA (-sscDNA) (137 bases)^a^		
Forward primer	oHC64	5′-TAACTAGGGAACCCACTGC
Reverse primer	oHC65	5′-GCTAGAGATTTTCCACACTG
Probe	oHC66	5′-FAM-ACACAACAGACGGGCACACACTA-TAMRA

EGFP (1426 bases)^a^		
Forward primer	EGFP.F	5′-GCGCACCATCTTCTTCAAGG
Reverse primer	EGFP.R	5′-GTGTCGCCCTCGAACTTCAC
Probe	EGFP.Probe	5′-FAM-CGGCAACTACAAGACCCGCGC-TAMRA

Late: Second strand transfer ((+)strand) (3958 bases)^a^		
Forward primer	oHC64	5′-TAACTAGGGAACCCACTGC
Reverse primer	GagM661as	5′-CTGCGTCGAGAGAGCTCCTCTGGTT
Probe	oHC66	5′-FAM-ACACAACAGACGGGCACACACTA-TAMRA

^a^The expected length of the corresponding reverse transcribed DNA is indicated in brackets

### Blocking RNase H activity inhibits uncoating

**Fig. 2:**
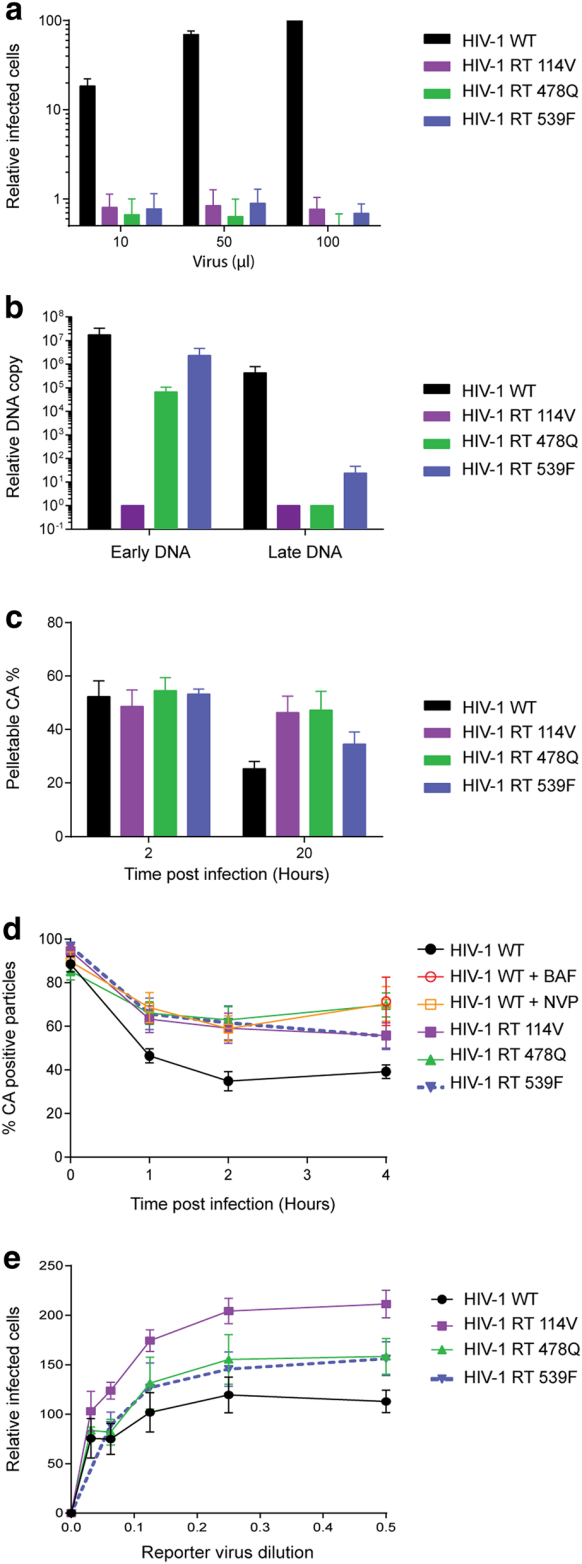
Effect of RT mutations on infectivity, reverse transcription and uncoating. **a**, **b**, **c** 293T cells were infected with either WT (*black*) GFP-HIV VLP or VLP carrying mutations in RT at A114V (*purple*), E478Q (*green*) or H539F (*blue*). **a** The percentage of GFP positive cells was measured by flow cytometry 72 h.p.i. with increasing amounts of VLP, and plotted relative to the number of cells infected by 100 μl WT VLP. **b** The levels of early and late cDNA were detected by qPCR 6 h.p.i. **c** Fate-of-CA assay. HeLa cells were infected with WT or RT mutant GFP-HIV VLP. Cells were lysed 2 or 20 h.p.i. and lysate separated into soluble and pellet fractions by centrifugation through a sucrose cushion. CA was detected by immunoblotting, quantified and the percentage of total CA in the pellet fraction is plotted. **d** In situ uncoating assay. HeLa cells were infected with dual-labeled WT or RT mutant HIV VLP in the presence (*orange*) or absence of 10 µM NVP and then fixed and stained for CA at the indicated times post-infection. Baf A (*red cirle*) was added to one sample as a negative control for fusion. The percentage of fused particles that still stain for CA was calculated 1, 2, or 4 h post-infection. **e** Saturation assay. Vero cells were infected with 2 fold serial dilutions of freshly harvested 293T cell supernatants containing WT or RT mutant LacZ-HIV VLPs. After 4 h, cultures were challenged with a fixed titre of WT GFP-HIV VLP. The percentage of GFP positive cells 72 h later is plotted relative to the infection seen following pre-infection with undiluted WT LacZ-HIV VLP. *Each panel* shows the mean and SEM of >3 independent experiments

First, we investigated whether uncoating begins early, before the synthesis of −sscDNA, by studying HIV-1 VLP carrying mutations in RT [22–26]. The A114V catalytic domain mutation inhibits polymerase activity of RT [24] while mutations E478Q and H539F induce a complete reduction or a 50 % decrease in RNase H activity in vitro respectively [23]. Equal p24 units of VLP were used to challenge 293T cells and the percentage of infected cells was measured 72 h post-infection (h.p.i.) by flow cytometry. All mutations reduced VLP infectivity to background levels (Fig. 2a). DNA was purified from infected cells at various times post-infection and the levels of early and late cDNA products were measured by qPCR. VLP carrying the A114V mutation failed to synthesise even −sscDNA, whilst the RNase H mutants were competent to synthesise early but not late cDNA (Fig. 2b), with E478Q more compromised than H539F. As these VLP were not infectious, we were unable to test the effects of these mutations on uncoating in the CsA washout assay. Instead, to investigate uncoating in a variety of ways and to limit the shortcomings of each, we performed four previously published assays; an in vitro core disassembly assay [15], a “fate-of-CA” assay [27], an in situ uncoating assay [28] and a TRIM5α abrogation assay [29]. In addition, these assays utilise multiple cell lines, removing any bias that may occur in any individual line.

First, for the in vitro core disassembly assay, isolated viral cores were incubated with dNTPs for various times to allow reverse transcription to occur. Soluble CA was then separated from core-associated CA by centrifugation through a sucrose cushion and the fractions were analysed by immunoblotting and densitometry. The percentage of CA in the pellet fraction was calculated and plotted (Additional file 1: Fig. S1). After 2 h, 50 % of the CA was detected in the pelletable fraction for all viruses, indicating that there was no difference in core disassembly of the mutants in vitro. To assess uncoating in vivo, we first used a fate-of-capsid assay. HeLa cells infected with WT HIV-1 or RT mutants were lysed after 2 or 20 h and CA complexes in the cell lysate were separated from soluble CA by centrifugation through a sucrose cushion (Additional file 1: Fig. S1). The percentage of CA in the pelletable fraction was calculated as a measure of the degree of uncoating that had occurred. Figure 2c shows that 20 h.p.i., only 25 % of CA in WT infections was pelletable, but 50 % of the CA from A114V or E478Q mutant VLP and ~35 % of CA from H539F was in the pellet fraction. Therefore, the amount of CA in the pellet inversely correlated with the amount of late cDNA products synthesised by each mutant (Fig. 2b). We next performed an in situ uncoating assay [28]. HIV-1 VLP were labelled with two fluorescent proteins: GFP-Vpr to follow viral cores, and S15-mCherry to identify viral membranes [30]. This allows discrimination of particles that have successfully fused with cell membranes from those that have not yet entered the cytoplasm. Bafilomycin A (Baf A) was added to one sample as a negative control for fusion. Following synchronised infections, cells were fixed and stained for CA at the indicated times and imaged. GFP, mCherry and CA puncta were identified and overlayed (Additional file 1: Fig. S1). All GFP positive, mCherry negative puncta were then classified as either CA positive (coated) or negative (uncoated) and the percentage of CA positive particles was quantified (Fig. 2d). The percentage of CA positive particles decreased with time for all infections but was most pronounced in WT infections where only 40 % of GFP positive particles were associated with CA after 2 h. The percentage of CA positive particles for all the RT mutants was similar to WT infections in the presence of either NVP or Baf A, suggesting that uncoating was similarly delayed for all of these mutants. Finally, we performed a TRIM5α restriction abrogation assay [29]. TRIM5α, like TRIMCyp, restricts incoming virions by binding to their CA shells [18], but lower endogenous expression levels compared to TRIMCyp enable high viral titres to saturate the protein and abrogate restriction. Once a virion has lost its CA shell, TRIM5α cannot bind, so uncoated particles cannot abrogate restriction. Vero cells expressing endogenous TRIM5α were infected with serial dilutions of WT or RT mutant LacZ-encoding HIV-1 VLP before being challenged with a fixed titre of WT GFP-encoding HIV-1 VLP. The number of cells expressing GFP was measured by flow cytometry after 48 h (Fig. 2e). Prior exposure of cells to the A114 V mutant VLP enhanced the GFP-encoding HIV-1 infectivity by 200 % compared to prior exposure to WT VLP, suggesting that the mutant VLP were better at saturating TRIM5α. Prior exposure to either RNase H mutant also enhanced the GFP-HIV-1 infectivity compared to WT, by 150 %. In summary, using four different uncoating assays, we observed that inhibiting RNAse H function stabilised the CA shell of HIV-1 particles, apparently delaying or preventing uncoating. Therefore, although blocking reverse transcription inhibits uncoating, early DNA synthesis can occur without triggering the uncoating process.

### Uncoating is not dependent on late products of reverse transcription

**Fig. 3:**
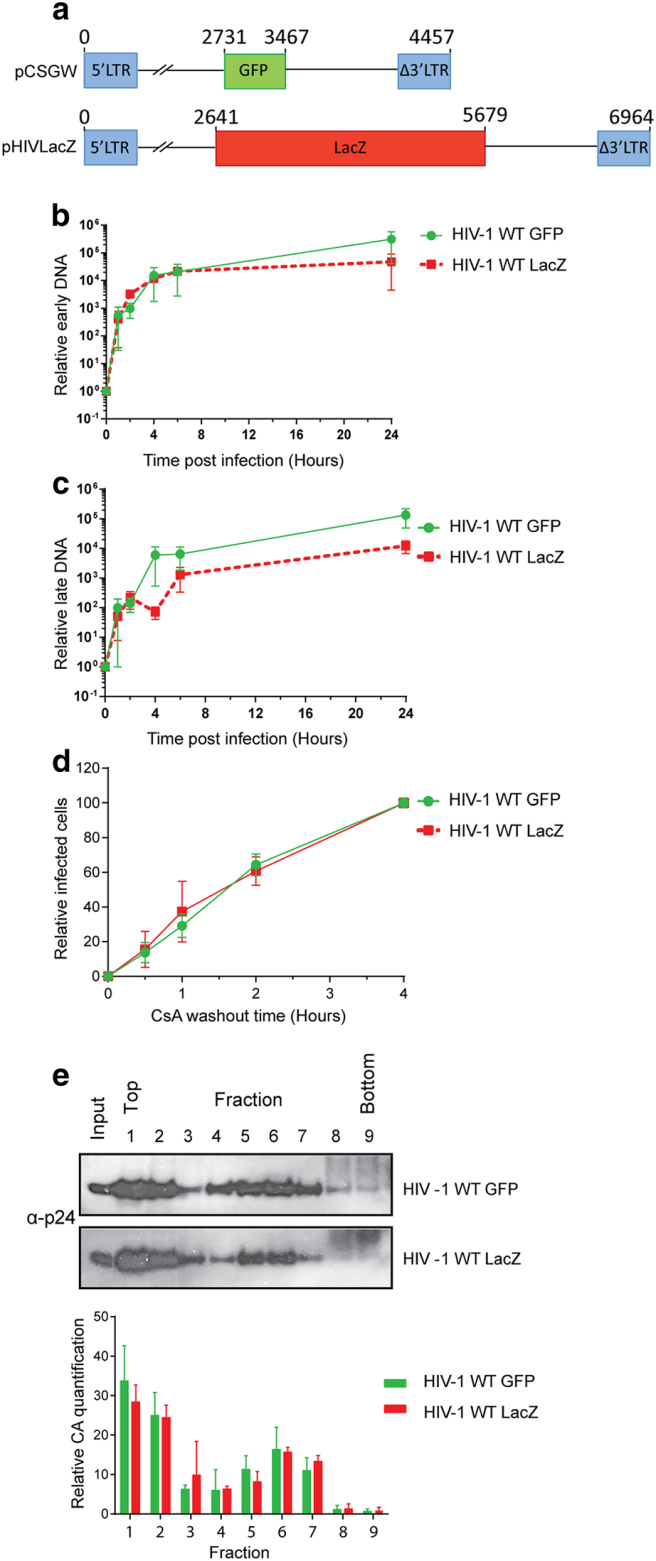
Effect of viral RNA length on reverse transcription and uncoating. **a** Schematic representation of pCSGW and pHIVLacZ plasmids highlighting key positions in bp. **b**, **c** 293T cells were infected with either WT GFP-HIV (*green*) or LacZ-HIV (*red*) VLP. At the indicated times post-infection, the level of cDNA corresponding to **b** early or **c** late reverse transcription products were analysed by qPCR. **d** CsA washout assay. OMK cells were infected with GFP-HIV or LacZ-HIV VLP in the presence of CsA. CsA was removed from each sample at the indicated times. Infection was measured after 72 h and plotted relative to that observed when CsA was removed 4 h.p.i. **e** Modified fate-of-CA assay. 293T cells were infected with GFP-HIV or LacZ-HIV VLP. Cells were lysed 4 h.p.i. and lysate separated in a 10–50 % (w/v) sucrose gradient. The gel shows a representative image from 3 biological repeats. The CA content in each fractions was detected by immunoblotting, quantified and the percentage of CA relative to the total cell lysate (input) was calculated and plotted. *Each graphical panel* shows the mean and SEM of at least 3 independent experiments

As it appeared that initiation of reverse transcription was not sufficient to induce uncoating, we then tested whether completion of reverse transcription was the trigger for uncoating. We compared the rates of uncoating for VLP with different sized genomes. The genomes were identical except that one coded for GFP and the other for LacZ, making them 4457 or 6964 base pairs long respectively (Fig. 3a). Although early reverse transcripts were produced at similar rates for the two particles (Fig. 3b), there was a delay in the accumulation of late (+)strand products for VLP containing the longer genomes (Fig. 3c). However, there was no difference in the rate of uncoating for these particles, as measured by the CsA washout assay (Fig. 3d) or a modified fate-of-capsid assay where infected cell lysates were separated in 10–50 % (w/w) sucrose gradients to assay for even small changes in CA complex size (Fig. 3e). This suggests that uncoating is independent of the length of the final cDNA product and the time taken to complete (−)strand synthesis.

### First strand transfer is required for uncoating

**Fig. 4:**
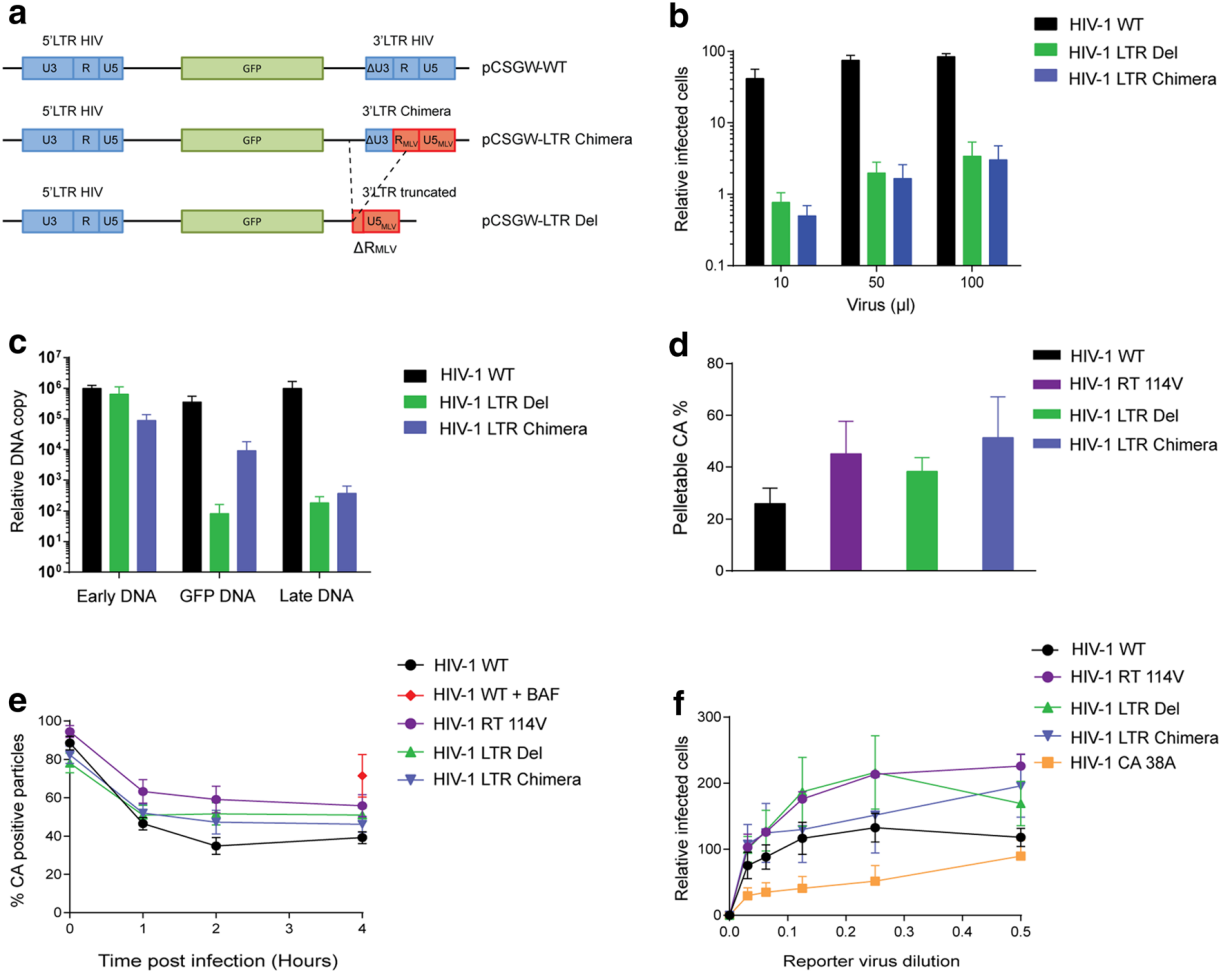
Effect of FST mutations on infectivity, reverse transcription and uncoating. **a** Schematic representation of FST mutants. **b**, **c** 293T cells were infected with either WT (*black*) GFP-HIV VLP, VLP carrying a chimeric (*blue*) or partially deleted (*green*) 3′LTR. **b** The percentage of GFP positive cells was measured by flow cytometry 72 h.p.i. with increasing amounts of VLP, and plotted relative to the number of cells infected by 100 μl WT VLP. **c** The levels of early, intermediate (GFP gene) and late cDNA were detected by qPCR 6 h.p.i. **d** Fate-of-CA assay. HeLa cells were infected with either WT (*black*) GFP-HIV VLP or VLP carrying the RT mutation A114V (*purple*), a chimeric 3′LTR (*blue*) or partially deleted 3′LTR (*green*). Cells were lysed 20 h.p.i. and lysate separated into soluble and pellet fractions by centrifugation through a sucrose cushion. CA was detected by immunoblotting, quantified and the percentage of total CA in the pellet fraction plotted. **e** In situ uncoating assay. HeLa cells were infected with dual-labeled WT or mutant HIV VLP and then fixed and stained for CA at the indicated times post-infection. Baf A (*red circle*) was added to one sample as a negative control for fusion. The percentage of fused particles that still stain for CA was calculated 1, 2, or 4 h.p.i. **f** Saturation assay. Vero cells were infected with 2 fold serial dilutions of freshly harvested 293T cell supernatants containing WT, mutant GFP-HIV VLPs or P38A capsid mutant GFP-HIV VLP (*orange*). After 4 h, cultures were challenged with a fixed titre of WT LacZ-HIV VLP. The level of β-galactosidase activity in cell lysates was measured after 72 h and is plotted relative to the level of infection seen following pre-infection with undiluted WT GFP-HIV VLP. *Each panel* shows the mean and SEM of at least 3 independent experiments

Our experimental data therefore suggest that uncoating is initiated after RNaseH degradation of RNA following −sscDNA synthesis but before second strand transfer. Four steps occur between these stages of reverse transcription: First strand transfer, first strand elongation, RNase H degradation and initiation of (+)strand synthesis. To assess whether first strand transfer was required for uncoating, we designed two viral mutants to be defective at this step (Fig. 4a). First, we synthesised an HIV-1 genome with a chimeric 3′LTR (pCSGW-LTR Chimera) by replacing the R-U5 region from the HIV-1 3′LTR with that of MLV, to retain transcription signals whilst removing repeat signals. Secondly, we removed U3 and part of R from pCSGW-LTR Chimera to create pCSGW-LTR Del. As expected, both mutants had severely impaired infectivity in single cycle infectivity assays (Fig. 4b) but were able to generate −sscDNA as well as WT VLP (Fig. 4c). Therefore, these mutations did not affect particle formation or initiation of reverse transcription. Surprisingly, the LTR chimera mutant was able to undergo first strand transfer and generate longer (−)strand transcripts coding for GFP, albeit not as efficiently as WT VLP (Fig. 4c). However, the levels of late cDNA were over 1000 times lower than in cells infected with WT VLP (Fig. 4c) suggesting that cumulatively, strand transfer was impaired in this mutant. First strand transfer was strongly inhibited in the LTR Del mutant (Fig. 4c). We then assessed the effect of these mutations on uncoating.

In the fate-of-CA assay (Additional file 2: Fig. S2; Fig. 4d), the percentage of pelletable CA was increased 150–200 % in cells infected with the LTR mutants compared to WT VLP, similar to the RT A114V mutant. Likewise, in the in situ uncoating assay (Fig. 4e), uncoating was delayed for both the LTR mutants compared to WT VLP, although the delay was not as great as seen with the RT A114V or RNase H mutants (Fig. 2d). Finally, in the TRIM5α restriction abrogation assay (Fig. 4f), prior exposure of vero cells to the LTR-Del mutant enhanced the infectivity of the HIV-1 reporter VLP by 200 % compared to WT VLP, similar to the RT A114V mutant, suggesting that this mutant is better at saturating TRIM5α than WT VLP. However, prior exposure to the LTR-chimera mutant did not markedly enhance reporter VLP infectivity (Fig. 4f). The assay was validated by showing that the P38A CA mutant previously reported to uncoat more rapidly than WT VLP [15] was unable to enhance reporter VLP infectivity as well as WT VLP (Fig. 4f). Taken together, these results suggest that blocking first strand transfer (mutant LTR-del) leads to delayed uncoating. Reducing first strand transfer (mutant LTR-chimera) delays uncoating in the in situ assay but only has a mild effect on TRIM5α abrogation. This implies that the trigger for uncoating is after first strand transfer. Experimentally, it is problematic to be more precise than this, as it is difficult to individually block first strand elongation or (+)strand synthesis. Therefore, we decided to develop a mathematical model to independently define the point of reverse transcription that triggers uncoating.

### Developing a mathematical model for reverse transcription

In order to develop a quantitative understanding of the kinetics of uncoating in relation to the kinetics of reverse transcription, we used the experimental observations from our CsA washout assays to parameterise a mathematical model and infer the putative position of an uncoating threshold. As our conclusions about the timing of uncoating did not incorporate these data and were based only on the results of our experiments using viral mutants, the modelling would therefore represent an independent assessment of the uncoating process.

**Fig. 5:**
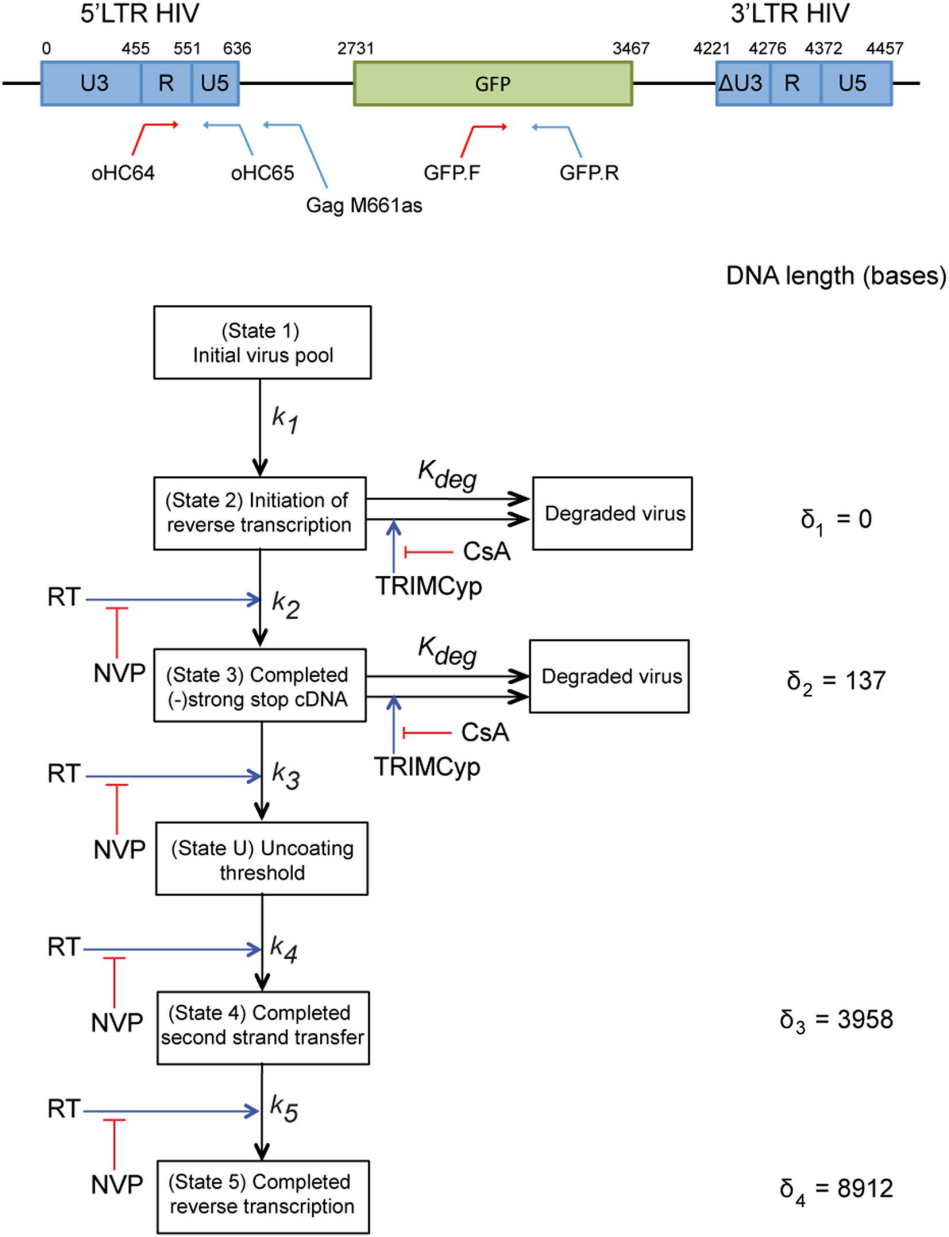
Schematic illustration of the mathematical model. *Top* shows a schematic representation of the pCSGW plasmid highlighting the position of primers used for the qPCR. *Bottom* illustrates the proposed six-state model of reverse transcription highlighting the effects of NVP and CsA. The lengths of the reverse transcribed cDNA corresponding to each state are indicated on the right

We developed a six state mathematical model (Fig. 5) that describes particle progression through reverse transcription. The states in the model represent various sequential reverse transcription landmarks, from initiation through to completion and are defined as follows: in State 1, particles are not yet transcriptionally active; in State 2, particles have initiated reverse transcription but not yet reverse transcribed −sscDNA $(\delta_{1} = 0\;{\text{bases}})$; in State 3, particles have reverse transcribed −sscDNA but not yet undergone second strand transfer ($\delta_{2} = 137$ bases); in State 4, particles have completed second strand transfer but not yet finished reverse transcription ($\delta_{3} = 3958\;{\text{bases}}$) and in State 5, transcription is complete ($\delta_{4} = 8912\;{\text{bases}}$).

To infer a putative position beyond which TRIMCyp loses efficacy, we introduced state U at an arbitrary position along the viral genome ($\delta_{1} < bp^{*} < \delta_{4}$) such that upon CsA removal, all particles in preceding states are immediately degraded. Qualitatively, we defined three alternative hypotheses for the timing of uncoating: (1) early ($\delta_{1} < bp^{*} < \delta_{2}$), (2) intermediate $(\delta_{2} < bp^{*} < \delta_{3} )$ and (3) late $(\delta_{3} < bp^{*} < \delta_{4} )$. For brevity, in the presentation of Fig. 5 and in the model equations below, we only show the outcome of uncoating occurring between the transcription of -sscDNA (State 3) and second strand transfer (State 4).

We assume that initiation of reverse transcription from a latent pool occurs at rate $k_{1}$ and transition rates between subsequent states are defined by the parameter $k_{i} .$ Once reverse transcription has initiated, we assume that the average time spent in each state is proportional to the number of base pairs that need to be transcribed to progress, i.e. the transition rates satisfy

$$ k_{i} = \frac{\ln \,(2)}{{ t_{bp} \left( {\delta \left[ i \right] - \delta \left[ {i - 1} \right]} \right).}}, \quad i > 1, $$

where $t_{bp}$ is the average time taken for a particle to reverse transcribe one base pair and $\delta = \left[ {\delta_{1} , \delta_{2} , bp^{*} ,\delta_{3} ,\delta_{4} } \right].$

To account for the intrinsic degradation of coated particles that occurs in host cells, we assume that in the absence of TRIMCyp, degradation occurs at background rate $k_{deg}$ and that in the presence of TRIMCyp degradation is concomitant with CA binding and instantaneous. As viral infectivity assays at different NVP concentrations yielded a value for the I_0_ constant *K*_*NVP*_ of 0.1 μM, in agreement with previous studies [31], we assumed an inverse dependence of reverse transcription rate parameters on NVP, i.e.

1 $$ g\left( {NVP\left( t \right)} \right) = \frac{1}{{1 + \frac{NVP\left( t \right)}{K_{NVP}}}}, $$

where $NVP\left( t \right)$ is specified in a given experiment. The complete model equations are given by

2 $$ \begin{aligned} \frac{{dN_{1} }}{dt} & = - k_{1} N_{1} , \\ \frac{{dN_{2} }}{dt} & = k_{1} N_{1} - k_{2} g\left( {NVP\left( t \right)} \right)N_{2} - k_{\deg } N_{2} , \\ \frac{{dN_{3} }}{dt} & = k_{2} g\left( {NVP\left( t \right)} \right)N_{2} - k_{3} g\left( {NVP\left( t \right)} \right)N_{3} -  k_{\deg } N_{3} , \\ \frac{{dN_{U} }}{dt} & = k_{3} g\left( {NVP\left( t \right)} \right)N_{3} - k_{4} g\left( {NVP\left( t \right)} \right)N_{U} , \\ \frac{{dN_{4} }}{dt} & = k_{4} g\left( {NVP\left( t \right)} \right)N_{U} - k_{5} g\left( {NVP\left( t \right)} \right)N_{4} , \\ \frac{{dN_{5} }}{dt} & = k_{5} g\left( {NVP\left( t \right)} \right)N_{4} , \\ \end{aligned} $$

with initial conditions representing the case of no initial reverse transcription products, i.e.

$$ N_{1} \left( 0 \right) = 100; \quad N_{i} \left( 0 \right) = 0,\quad i > 1. $$

To identify the four unknown model parameters, we used the data presented in Fig. 1c–f, where a series of experiments were performed that measured variables that are directly comparable with quantities described by the model. The −sscDNA and (+)strand products of reverse transcription were measured following different treatments with the reverse transcription inhibitor Nevirapine, and viral infectivity was measured against various backgrounds of Nevirapine and CSA.

**Fig. 6:**
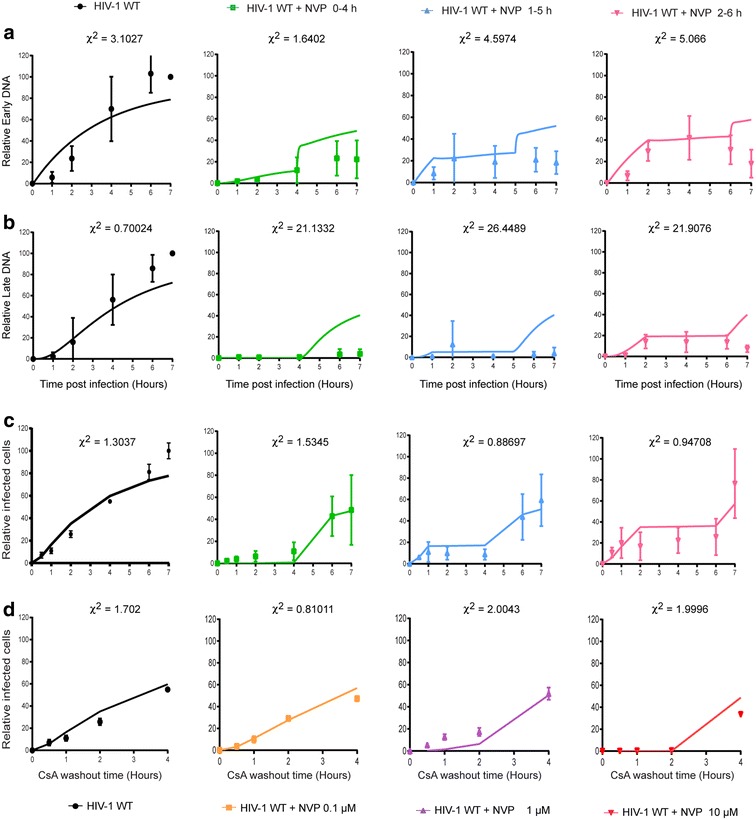
Modelling HIV-1 uncoating relative to reverse transcription. For each panel, points represent experimental data and *solid lines* indicate model solutions. **a**, **b** 293T cells were infected with GFP-HIV VLP in the presence of 10 μM NVP for 4 h starting at 0, 1 or 2 h.p.i. Early (**a**) and late (**b**) cDNA was analysed by qPCR. **c**, **d** CsA washout assays. OMK cells were infected with GFP-HIV VLP in the presence of **c** 10 μM NVP for 4 h starting at 0, 1 or 2 h.p.i. or **d** increasing concentrations of NVP for 0–2 h. CsA was removed from each sample at the indicated time. After 72 h the percentage of GFP positive cells was measured and plotted relative to the percentage of GFP positive cells following infection in the absence of NVP when CsA was removed 7 h.p.i. *Each panel* shows the mean and SD of at least 3 independent experiments. Model solutions were computed using Eq. (2) stated in the text and the best fit parameters $k_{1} = 0.29\,{\text{h}}^{ - 1}$; $t_{bp} = 2.4 \times 10^{ - 4} \,{\text{h}}$; $k_{deg} = 0.3\,{\text{h}}^{ - 1}$; $bp^{*} = 974$ bases. Other parameters were: *K*_*NVP*_ = 0.1 μM, N_init_ = 100, bp_–sscDNA_ = 137; bp_(+)strand_ = 3958

**Fig. 7:**
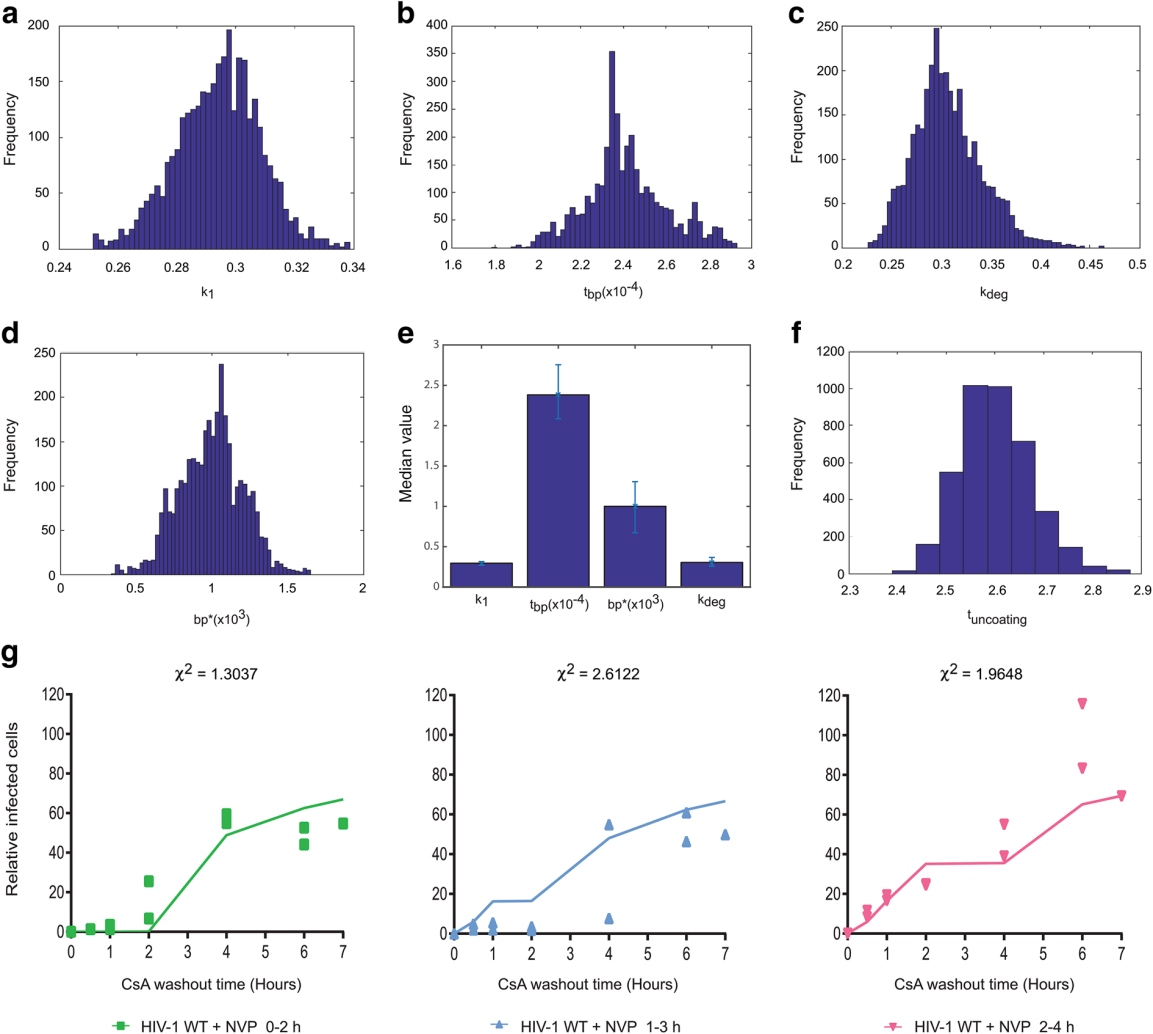
Robustness of parameter fitting and testing the mathematical model of uncoating. **a**–**d** Estimated marginal distributions were computed for each model parameter from the calculated posterior distribution. Best fit parameters were defined using median values of marginal distribution and presented with 95 % credible intervals in (**e**). **f** The time of uncoating, t_uncoating_ was estimated using the best fit parameters. **g** Testing the model predictions of infection kinetics in the CsA washout assay. OMK cells were infected with GFP-HIV VLP in the presence of CsA and 10 μM NVP for 2 h starting at 0, 1 or 2 h.p.i. CsA was removed from each sample at the indicated time. After 72 h, the percentage of GFP positive cells was measured for each sample and plotted relative to the percentage of GFP positive cells following infection in the absence of NVP when CsA was removed 7 h.p.i. *Points* represent experimental data from two independent experiments and *solid lines* indicate model predictions

Using Bayesian inference, we identified regions of parameter space that best fit the experimental data (see Fig. 6 for best fit solutions and Fig. 7a–e for parameter robustness estimates). The value of $k_{1}$ that best fits the data is $0.29 \pm 0.02\,{\text{h}}^{ - 1}$. The value of $t_{bp}$, the average time taken to transcribe one base pair, is $2.4 \pm 0.37 \times 10^{ - 4} \,{\text{h}}$, a value that is similar to previously reported values. We find that the parameter $k_{deg}$ is $0.3 \pm 0.06\,{\text{h}}^{ - 1}$, suggesting that even in the presence of CSA, the half life of particles is of the order of 2 h. Finally, we estimate that $bp^{*} = 974 \pm 326$ bases (errors represent 95 % credible intervals). Within our GFP vector, the uncoating threshold is reached on the negative strand just before the sequence coding for GFP, while in the HIV-1 complete genome it corresponds to the sequence ~50 bp upstream of the Nef coding region, which is ~10 % along the total transcribed genome. Therefore, as uncoating must be triggered before State U, it is likely to start soon after first strand transfer, as (−)strand cDNA extends or, perhaps more likely, as (+)strand synthesis initiates and creates a short section of double stranded (ds)DNA. Using the fitted parameters to calculate the average time spent in state 1 (2.35 h), and adding to this the time taken to reverse transcribe the 1000 bases necessary to get to the threshold (0.24 h), our model kinetics estimate uncoating to occur $2.6 \pm 0.13$ h.p.i. (Fig. 7f). This is similar to recent reports of the timing of uncoating [21]. Finally, to independently test the fitted model, we performed additional CsA washout experiments in which NVP was applied for 2 h windows, and compared the model fits to experimental data (Fig. 7g). The calculated Chi squared measurements show that the model was able to satisfactorily predict the experimental outcomes, leading us to conclude that the predicted timing of uncoating was a reasonable estimation.

In summary, by fitting the model to infectivity data from CsA washout experiments carried out under a range of NVP concentrations, we predict that virus is sensitive to TRIMCyp until some time after the transcription of -sscDNA. This is in excellent agreement with our conclusions from experiments with viral mutants in which we blocked specific steps of reverse transcription. In turn, this suggests that uncoating is not triggered merely by the initiation of reverse transcription, and also lends support to the idea that late reverse transcription occurs after uncoating has begun.

## Discussion

Understanding the timing and triggers for uncoating has become central to investigations of all aspects of the early stages of retroviral replication. Here, we sought to identify the point of reverse transcription that initiates the uncoating process to determine how reverse transcription and uncoating are linked. We generated experimental data using various viral mutants to inhibit reverse transcription at several specific points, and also developed and parameterised a mathematical model for reverse transcription that could investigate the effect of NVP on uncoating in a CsA washout assay. Both approaches came to similar conclusions.

As there is no definitive assay for CA uncoating, we tested the effects of our viral mutations on uncoating using a range of uncoating assays (Figs. 2, 4). Each assay has its limitations, for example, in vitro assays are performed in the absence of cellular factors that may contribute to the uncoating process. Furthermore, biochemical and microscopic assays, such as the fate-of-CA and in situ uncoating assay, monitor bulk populations of virus particles, many of which may be defective and may exhibit misleading behaviours. The timing of the measurements post-infection also affects the outcomes of these assays. Although the CsA washout assay has the advantage that it only measures the effects on particles that go on to form productive infections, it is indirect and uses TRIMCyp restriction as a readout, which is itself poorly understood. It is not known how many CA molecules must be present on particles for TRIMCyp to restrict infection, although as TRIMCyp binds capsid shells very soon after viral entry, it seems likely that the CsA washout assay measures the early stages of uncoating. Another disadvantage of this assay is that non-infectious mutants like our RNase H mutants cannot be tested. For this reason, we repurposed a similar assay, the TRIM5α abrogation assay, to test capsid shell integrity in yet another way. However, despite the differences between all the assays used, the data from all our analyses were in general agreement, giving us conviction to conclude that uncoating is triggered by a mid point in reverse transcription, between the two strand transfers.

Experimentally, it is difficult to be more specific about the point at which uncoating is triggered as it is not possible to individually block first strand elongation or second strand synthesis. However, using our mathematical model, derived from independent experimental data, we estimated the point of reverse transcription at which uncoating occurs. We introduced an additional state into the model to constrain the point at which TRIMCyp loses efficacy and determined, by minimising the error between model and experimental observations, that the optimal position for it was approximately 1000 bases along the viral genome (Fig. 7). Therefore, uncoating is likely to start at an early step after first strand transfer as it must be triggered before this additional state. As with the experimental data, the model has some limitations. Whilst it is in reasonable agreement with the infected cell data, it does not fully capture the qPCR measurements following NVP removal. The reason that the model over-estimates the recovery of reverse transcription after NVP treatment is unclear and may reflect off-target effects of the drug or unaccounted for cellular influences on the virus.

The fact that both analysis of viral mutants and an independent mathematical model suggest uncoating occurs at this stage of reverse transcription does, however, give us confidence in our conclusions. Specifically, we propose that uncoating is triggered either by elongation of the (−)strand viral cDNA, or by initiation of (+)strand synthesis to generate a dsDNA fragment. Either scenario would increase the bulk of DNA in the particle, and the more structured dsDNA may pack less efficiently inside the capsid shell. Therefore, uncoating could be caused by the increasing volume constraint on the capsid. Alterations in CA that influence the intrinsic stability of the shell or modulation of the interactions of cellular factors with CA could affect the resistance of the shell to such force, and affect the timing of uncoating [11, 12, 32]. This could result in the loss of some capsid without necessitating the loss of all of the capsid from the PIC, fitting with recent reports of a bimodal dissociation of CA [33, 34]. However, dsDNA might also be detected by a specific mechanism leading to programmed uncoating. Interestingly, early access to the viral core is reportedly not affected by reverse transcription [34], although this did not correlate with overall CA shell stability or the major loss of CA.

Taken together with reports that CA may be present in the nucleus [33, 35] and influences nuclear replication steps such as integration [36, 37], we can perhaps consider the uncoating process in three phases: (1) Early opening of the CA lattice allowing entry of cellular factors such as dNTPs and other molecules required for reverse transcription. (2) The loss of the majority of CA and the integrity of the lattice. (3) The loss of the remaining CA inside or at the nucleus. Phase one could be initiated by entry of the viral core into the cytoplasm. Phase 2 triggered during reverse transcription, and phase 3 accomplished upon integration of the viral cDNA. Therefore, the general term “uncoating” is probably most applicable to phase 2. Notably, loss of the majority of CA before completion of reverse transcription suggests that the CA shell alone is not responsible for protecting the viral DNA from innate immune sensing. However, if phase 2 of uncoating occurred at the nuclear periphery, then other factors may ‘cloak’ the viral DNA until reverse transcription is finished. Alternatively, reverse transcription could be completed inside the nucleus. Further work is needed to elucidate the role of CA in nuclear import and integration, but it is clear that the timing of the bulk of CA loss is linked to replication of the viral genome.

## Conclusions

HIV-1 CA uncoating is very difficult to study because it is a dynamic process and direct observation is obscured by the presence of non-infectious particles and issues with sensitivity of detection. However, it is important to understand the behaviour of the CA shell as it appears to be critical for several post-entry steps during replication. By combining experimental data from multiple uncoating assays with a novel mathematical model for uncoating, we aimed to identify the point in reverse transcription that triggers uncoating. We show here that uncoating is triggered following first strand transfer and propose that either elongation of the (−)strand viral cDNA, or initiation of (+)strand synthesis is sufficient to start the breakdown of the HIV-1 CA shell.

## Methods

### Cells

293T, HeLa, OMK and VERO cells (authenticated, mycoplasma-free Bishop laboratory cell stocks) were maintained in Dulbecco’s modified Eagle medium (Invitrogen) supplemented with 10 % heat-inactivated foetal bovine serum (Biosera) and 1 % Penicillin/Strepomycin/Glutamine (Sigma). Cyclosporine A (CsA; Sigma) was prepared in DMSO at 300 μM, and used at a final concentration of 3 μM. Nevirapine (NVP; National Institutes of Health (NIH) AIDS Research and Reference Reagent Program) was prepared in ETOH at 1 mM and used at a final concentration of 0.1; 1 or 10 μM. Bafilomycine A1 (Sigma) was prepared in DMSO at 2 mM, and used at a final concentration of 20 nM.

### Plasmids and cloning

HIV-1 VLP were produced by cotransfection of pVSV-G, pCMVΔ8.91 and either pCSGW or pWPTS-nlsLacZ [38]. The pCMVΔ8.91 construct expressing RT mutant A114V was gift from J. Stoye. To create the other mutant Gag-Pol plasmids, site directed mutagenesis was performed on pCMVΔ8.91 using the Quik-Change kit (Stratagene) using the following primers; RNase H mutant E478Q: *for* 5′-*CACAACAAATCAGAAGACTCAGTTACAAGCAATTCATCTA* and *rev* 5′-*TAGATGAATTGCTTGTAACTGAGTCTTCTGATTTGTTGTG;* RNase H mutant H539F: *for* 5′-*CCTGGCATGGGTACCAGCATTCAAAGGAATTGGAGGAAAT* and *rev* 5′-*ATTTCCTCCAATTCCTTTGAATGCTGGTACCCATGCCAGG;* CA mutant P38A: *for* 5′-*GGCTTTCAGCCCAGAAGTGATAGCCATGTTTTCAGCATTA* and *rev* 5′-*TAATGCTGAAAACATGGCTATCACTTCTGGGCTGAAAGCC*. The LTR Chimera strand transfer mutant was constructed by overlapping PCR from three DNA fragments amplified from either pCSGW (for HIV ΔU3 and flanked region) or pczLTR-LacZ (for Mo-MLV R-U5 [39]). All DNA fragments were amplified using Phusion DNA polymerase (Finnzymes) with the following primers: *HIV ΔU3*: for 5′-*CGTCCCTTCGGCCCTCAATC* and rev 5′-*CTATCGGAGGACTGGCGCCCAGTACAAGCAAAAAGC*; Mo-MLV R-U5: for 5′-*GCTTTTTGCTTGTACTGGGCGCCAGTCCTCCGATAG* and rev 5′-*CGGAATTAATTCTAGATGCGCTGACGGGTAGTCAATC*; HIV-1 flanking region: for 5′-*GATTGACTACCCGTCAGCGCATCTAGAATTAATTCCG* rev 5′-*GCACCATTCGACGCTCTCCC*. The final PCR fragments were amplified with primers *for* 5′-*CGTCCCTTCGGCCCTCAATC* and *rev* 5′-*GCACCATTCGACGCTCTCCC*, and inserted into pCSGW between the *SacII* and *AfeI* restriction sites. The MLV 3′LTR partial deletion mutant was synthesised by digestion of the LTR Chimera mutant with KpnI.

### VLP production

HIV-1 VLP were synthesised by co-transfection of 293T cells with pCMVΔ8.91 (or mutants), VSV-G, and either pWPTS-nlsLacZ (for LacZ-expressing VLP) or WT or mutant pCSGW (for GFP-expressing VLP). After 24 h, cells were treated with 10 mM sodium butorate, and virus-containing supernatants were harvested 24 h later. For the in situ uncoating assay, dual-labeled VLP were produced by transfecting a 4:4:4:4:1 ratio of pCMVΔ8.91 (or mutants), pWPTS-nlsLacZ, VSV-G, pS15-mCherry and pGFP-VPR [30]. Virus-containing supernatants were harvested 24 h after transfection. All supernatants were purified through a 0.22-μM filter and titred using a p24 ELISA assay (PerkinElmer).

### Infectivity assay

293T cells were challenged with equivalent p24 units of GFP-HIV or LacZ-HIV VLP for 72 h. For GFP-VLP, the percentage of cells expressing GFP was determined by flow cytometry using a FACS VERSE analyser (Becton–Dickinson). Cells infected with LacZ-VLP were lysed and β-galactosidase activity in cell lysates was measured using the Galacto-Star system (Applied Biosystems).

### Quantitative PCR analysis

The quantitative PCR analysis was conducted as previously described [40]. Prior to infection, VLP were treated with 20 units/ml RQ1-DNase (Promega) in 10 mM MgCl_2_ for 1 h at 37 °C. 293T cells (2 × 10^5^) were spinoculated (1600×*g* at 16 °C for 30 min, followed by 37 °C for 30 min) and the media was replaced by warm DMEM. Cells were harvested at the indicated time point post-infection and total DNA was extracted using the DNeasy Blood & Tissue Kit (Qiagen). After digestion with 1 unit/μl DpnI for 1 h at 37 °C, 20 ng of DNA was analysed in triplicate (technical repeats) by qPCR using the iCycler iQ real-time PCR detection system (BioRad) with 900 nM primers and 250 nM probes (Table 1). The PCR reactions were performed on a Fast 7500 PCR system (Applied Biosystems) using standard cycling conditions: 50 °C for 2 min, 95 °C for 10 min followed by 40 cycles of 95 °C for 15 s and 60 °C for 1 min. Relative cDNA copy numbers were calculated using a standard curve generated from serial dilutions of pCSGW or pWPTS-nlsLacZ in 293T cellular DNA. Unless stated, graphs show the mean and SEM of at least 3 biological repeats.

### CsA washout assay

The CsA washout assay was previously described [9]. OMK cells endogenously expressing TRIMCypA were seeded at 1 × 10^5^ cells per well in 24-well plates 1 day prior to infection. Cells were spinoculated (1600×*g* at 16 °C for 1 h, followed by 37 °C for 30 min) with GFP or LacZ VLP in the presence of 3 μM CsA (Sigma) and 5 μg/ml polybrene with or without NVP (NIH AIDS Research and Reference Reagent Program; 0.1, 1 or 10 μM in EtOH). Inoculation media was exchanged for warm media containing CsA and/or NVP as appropriate, and this was considered the zero time point. CsA was removed at the indicated times post-infection by media exchange. NVP was added to the warm media at the indicated times and removed from all reactions by media exchange. Three days after infection, the percentage of GFP-positive cells was determined by flow cytometry. For cells infected with LacZ-VLP, cells were lysed and β-galactosidase activity in cell lysates was measured using the Galacto-Star system (Applied Biosystems). Unless stated, graphs show the mean and SEM of at least 3 biological repeats.

### In vitro core disassembly assay

Core isolation and in vitro disassembly were previously described [15]. VLP-containing supernatant from transfected 293T cells was concentrated through a 20 % (w/w) sucrose cushion at 32,000 rpm at 4 °C for 4 h in a Beckman SW32 rotor. Viral pellets were re-suspended in 250 μl PBS and centrifuged (28,500 rpm in a Beckman SW41 rotor at 4 °C for 16 h) through a layer of 1 % triton X-100 overlaying a 30 % (w/w) sucrose cushion to isolate viral cores. Core-containing pellets were re-suspended in 300 μl of PBS and incubated at 37 °C with 1 mM dNTPs and 50 mM MgCl_2_. At the indicated times post-incubation, samples were removed and centrifuged (32,000 rpm in a Beckman SW55 rotor at 4 °C for 1 h) through a 30 % (w/w) sucrose cushion. The top layer above the sucrose cushion was collected as the “soluble” fraction and once the remaining sucrose was removed, SDS-PAGE loading dye was added to the tube to re-suspend the “pellet” fraction. Fractions were analysed for CA protein content as in the fate-of- CA assay.

### Fate-of-capsid assay

The fate-of-capsid assay was previously described [27]. HeLa cells were seeded at 10^6^ cells/well in 6-well plates 1-day before infection and spinoculated (1600×*g* at 16 °C for 30 min, followed by 37 °C for 1 h) with 1.5 mL/well of concentrated GFP-VLP supernatant containing 5 μg/ml polybrene. Inoculation media was then replaced with warm DMEM. Cells were harvested 2 or 20 h.p.i. and cells from 6 wells were pooled for each infection. Cells were washed in PBS and resuspended in 7 mg/ml pronase (Sigma) before being pelleted and resuspended in 700 μl hypotonic buffer (10 mM Tris–Cl pH 8.0, 10 mM KCl, 1 mM EDTA supplemented with complete protease inhibitors (Roche)). After incubation on ice for 15 min, cell suspensions were applied to a Qiashredder column (Qiagen) and centrifuged at 20,000×*g* for 2 min at 4 °C. Cell lysates were layered on top of either a 10–50 % (w/v) linear sucrose gradient or on top of 30 % (w/w) sucrose cushion and centrifuged at 32,000 rpm for 1 h at 4 °C in a Beckman SW41 rotor. An aliquot of the cell lysate was kept as an input control. Nine 500 μl fractions were collected from the top to the bottom of the gradient using a syringe pump-driven gradient fractionator (Brandel). Proteins were extracted using methanol/chloroform and resuspended in SDS-PAGE loading dye. The top layer above the sucrose cushion was collected as the “soluble” fraction and once the remaining sucrose was removed, SDS-PAGE loading dye was added to the tube to re- suspend the “pellet” fraction. Fractions were analysed for CA protein content by immunoblotting using a mouse monoclonal anti-HIV-1 CA antibody, 24-2 (a gift from Michael Malim) followed by rabbit anti-mouse HRP-conjugated secondary antibody (Life Technologies Ltd). Detection was performed using the Immobilon chemiluminescent substrate (Millipore) and hyperfilm processed through a Fijifilm FPM-3800A developer. Quantification of band density was performed using Fiji software after scanning immunoblot on an HP Scanjet 3800.

### In situ uncoating assay

The in situ uncoating assay was conducted as previously described [28]. At least two different preparations of each virus were used for each assay. HeLa cells were seeded at 10^6^ cells/well on 13 mm coverslips in 12-well plates 1-day before infection and were spinoculated (1600×*g* at 16 °C for 1 h) with dual-labelled VLP. Inoculation media was replaced by warm DMEM and at the indicated time points post-infection, cells were fixed in 4 % paraformaldehyde (ChemCruz) for 20 min at room temperature. Fixed cells were then permeabilized with 0.1 % triton-100 for 10 min, washed with PBS, and blocked with 5 % normal donkey serum (NDS, Source Bioscience) for 1 h. Cells were then incubated in primary anti-CA antibody (p24 05-009, diluted 1/250 in 1 % NDS) overnight followed by 2 h incubation with donkey anti-mouse secondary antibody coupled to Alexa Fluor^647^ (Invitrogen) diluted 1:500. Samples were washed with PBS, mounted on slides and dried. Images were acquired using a Delta Vision Deconvolution microscope (Olympus IX70) and SoftWorks software (Applied Precision). Ten images were analysed for each viral infection at each time point. All GFP-Vpr positive puncta over 15.8 μm were counted using CellProfiler software (http://www.cellprofiler.org**)** and scored as associated or not with S15-mCherry and/or CA positive or negative. For each infection, the percentage of CA positive particles at the 0 h time point is the percentage of mCherry positive, CA positive, GFP positive puncta out of the total number of GFP positive puncta, and the percentage of CA positive particles at the remaining time points is the percentage of mCherry negative, CA positive, GFP positive puncta out of the total number mCherry negative, GFP positive puncta.

### Saturation assay

TRIM5alpha saturation assays were conducted as previously described [29]. Vero cells expressing endogenous TRIM5alpha were seeded at 10^5^ cells/well in 24-well plates one day prior to infection. Cells were infected with 2 fold serial dilutions of freshly harvested 293T cell supernatants containing GFP or LacZ-encoding VLP. Cultures were incubated for 4–6 h at 37 °C before adding a fixed amount of LacZ or GFP-encoding HIV-1 respectively (equivalent to an MOI of 0.5 in 293T cells). After 72 h, infected cells were harvested and either the percentage of GFP-positive cells was determined by flow cytometry or β-galactosidase activity in cell lysates was measured using the Galacto-Star system as for infectivity assays.

### Mathematical model

Matlab codes used to simulate the model can be found at https://github.com/murrayp/RTUncoatingModel.

#### Six state model

To compare the model with experimental measurements, Eq. (2) were solved numerically. The variable corresponding to measurable −sscDNA is given by the cumulant

$$ \varphi_{3} \left( t \right) = N_{U} \left( t \right) + \sum\limits_{i = 3}^{5} {N_{i} \left( t \right)} , $$

that corresponding to (+)strand cDNA by the cumulant

$$ \varphi_{5} \left( t \right) = \sum\limits_{i = 4}^{5} {N_{i} \left( t \right)} , $$

and the number of infected cells to be

$$ \varphi_{\infty } = N_{5} \left( {t \to \infty } \right). $$

#### Parameter inference

We define the likelihood for the strong stop dataset to be

$$ L_{1} = \prod\limits_{j = 1}^{{M_{1} }} {\prod\limits_{i = 1}^{{N_{1} }} {e^{{\frac{{ - \left( { \varphi_{j3} \left( i \right) - {{\widehat\varphi_{j3} }}\left( i \right)} \right)^{2} }}{{\sigma_{j3} \left( i \right)^{2}        }}}} } } , $$

the likelihood for the second strand dataset to be

$$ L_{2} = \prod\limits_{j = 1}^{{M_{2} }} {\prod\limits_{i = 1}^{{N_{2} }} {e^{{\frac{{ - \left( { \varphi_{j5} \left( i \right) - {{\widehat\varphi_{j5} }}\left( i \right)} \right)^{2} }}{{\sigma_{j5} \left( i \right)^{2}        }}}} } } , $$

and the likelihood for the infected cell dataset to be

$$ L_{3} = \prod\limits_{j = 1}^{{M_{3} }} {\prod\limits_{i = 1}^{{N_{3} }} {e^{{\frac{{ - \left( { \varphi_{j\infty } \left( i \right) - {{\widehat \varphi_{j\infty } }}\left( i \right)} \right)^{2} }}{{\sigma_{j\infty } \left( i \right)^{2}        }}}} } } , $$

where $$ M_{1} $$$$ M_{2} $$ and $$ M_{3} $$ represent different experiments, $$ N_{1} $$$$ N_{2} $$ and $$ N_{3} $$ the number of time points, hatted variables represent corresponding experimental measurement and the $$ \sigma_{js} $$ are sample standard deviations. The total likelihood is defined to be $$ L = L_{1} L_{2} L_{3} . $$

We define uniform (uninformative) priors on the four unknown parameters $\left( {U_{{\left[ {0.05,0.5} \right]}} , U_{{\left[ {1 \times 10^{ - 6} ,1 \times 10^{ - 2} } \right]}} , U_{{\left[ {0,10000} \right]}} , U_{{\left[ {1 \times 10^{ - 4} ,0.8} \right]}} } \right)$ and use slice sampling [41] to approximate the posterior distribution, given Baye’s Rule

$$ P(k_{1} t_{bp} bp^{*} k_{deg} |\theta ) = L(\theta |k_{1} t_{bp} bp^{*} k_{deg} )P\left( {k_{1} } \right)P\left( {t_{bp} } \right)P\left( {bp^{*} } \right)P\left( {k_{deg} } \right), $$

where $$ L(\theta |k_{1} t_{bp} bp^{*} k_{deg} ) $$ is the likelihood of the data, $$ \theta , $$$$ P\left( x \right) $$ represents prior probability distributions and $$ P(k_{1} t_{bp} bp^{*} k_{deg} |\theta ) $$ represents the posterior distribution.

To assess goodness of fit we calculated the Chi squared statistic

$$ \chi^{2} = \frac{1}{N}\sum\limits_{i = 1}^{N} {{{\left( {\varphi \left( i \right) - \widehat{\varphi }\left( i \right)} \right)^{2} } \mathord{\left/ {\vphantom {{\left( {\varphi \left( i \right) - \widehat{\varphi }\left( i \right)} \right)^{2} } {\sigma_{j} \left( i \right)^{2} }}} \right. \kern-0pt} {\sigma_{j} \left( i \right)^{2} }}} $$

In infected cell experiments in Fig. 2 (rows 3 and 4) there were only two measurements at each time point and the sample standard deviations from experiment in Fig. 2 (row 2) were used to compute Chi squared statistic .

## Additional files

10.1186/s12977-016-0292-7 Effect of RT mutations on capsid stability in vitro and on uncoating. **(a,b)** Isolated cores from either WT GFP-HIV VLP (black) or VLP carrying mutations in RT at A114 V (purple), E478Q (green) or H539F (blue) were incubated at 37 °C for 0, 1 or 2 h. Pelletable material was separated from soluble proteins by centrifugation through a sucrose cushion and CA was detected by immunoblotting. **(a)** A representative immunoblot from 3 independent experiments. **(b)** The CA bands in each immunoblot were quantified and the percentage of total CA in the pellet fraction was calculated. The graph shows the mean and SEM of 3 independent experiments. **(c)** Immunoblot of a fate-of-CA assay. HeLa cells were infected with WT or RT mutant GFP-HIV VLP. Cells were lysed 2 or 20 h.p.i. and lysate [input, I] separated into soluble [S] and pellet [P] fractions by centrifugation through a sucrose cushion. CA was detected by immunoblotting. Data is representative of 5 independent experiments. **(d)** Confocal microscopy images from an in situ uncoating assay. Left: HeLa cells infected with dual-labelled WT HIV-1 VLP were imaged 1 h.p.i. The outlined box is enlarged in the other three panels to show the distribution of S15-mCherry (red, denoting enveloped particles), Vpr-GFP (green, denoting viral cores), and CA (white). Fused virions are punctate spots that are GFP positive and mCherry negative, and were classified as associated with p24CA (horizontal arrows) or not associated with p24CA (vertical arrows).
10.1186/s12977-016-0292-7 Effect of FST mutations on capsid stability. **(a-b)** HeLa cells were infected with WT GFP-HIV VLP, or VLP carrying LTR mutations (LTR Del or LTR Chimera). Cells were lysed **(a)** 2 h or **(b)** 20 h.p.i. and cell lysate [input, I] was separated into soluble [S] and pellet [P] fractions by centrifugation through a sucrose cushion. CA was detected by immunoblotting. Blots are representative of 3 independent experiments.
