# Supplementary figures and images for: HIV-1 capsid uncoating initiates after the first strand transfer of reverse transcription
Source: Retrovirology. 2016 Aug 22;13(1):58. doi: 10.1186/s12977-016-0292-7 (PMC4994286; doi:10.1186/s12977-016-0292-7)

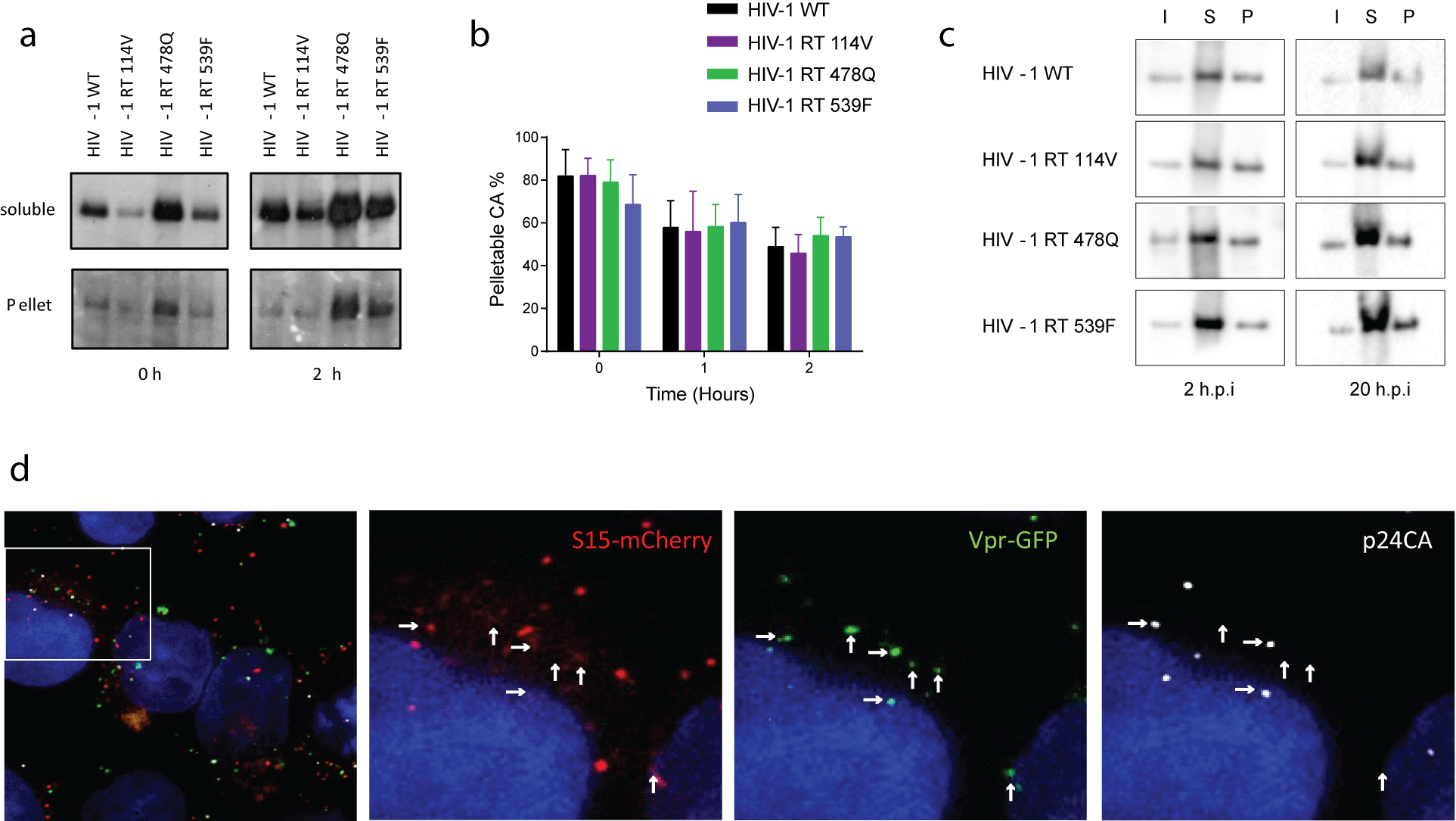

Supplement: Supplementary file 1 — 10.1186/s12977-016-0292-7 Effect of RT mutations on capsid stability in vitro and on uncoating. (a,b) Isolated cores from either WT GFP-HIV VLP (black) or VLP carrying mutations in RT at A114 V (purple), E478Q (green) or H539F (blue) were incubated at 37 °C for 0, 1 or 2 h. Pelletable material was separated from soluble proteins by centrifugation through a sucrose cushion and CA was detected by immunoblotting. (a) A representative immunoblot from 3 independent experiments. (b) The CA bands in each immunoblot were quantified and the percentage of total CA in the pellet fraction was calculated. The graph shows the mean and SEM of 3 independent experiments. (c) Immunoblot of a fate-of-CA assay. HeLa cells were infected with WT or RT mutant GFP-HIV VLP. Cells were lysed 2 or 20 h.p.i. and lysate [input, I] separated into soluble [S] and pellet [P] fractions by centrifugation through a sucrose cushion. CA was detected by immunoblotting. Data is representative of 5 independent experiments. (d) Confocal microscopy images from an in situ uncoating assay. Left: HeLa cells infected with dual-labelled WT HIV-1 VLP were imaged 1 h.p.i. The outlined box is enlarged in the other three panels to show the distribution of S15-mCherry (red, denoting enveloped particles), Vpr-GFP (green, denoting viral cores), and CA (white). Fused virions are punctate spots that are GFP positive and mCherry negative, and were classified as associated with p24CA (horizontal arrows) or not associated with p24CA (vertical arrows). [file 12977_2016_292_MOESM1_ESM.tif]

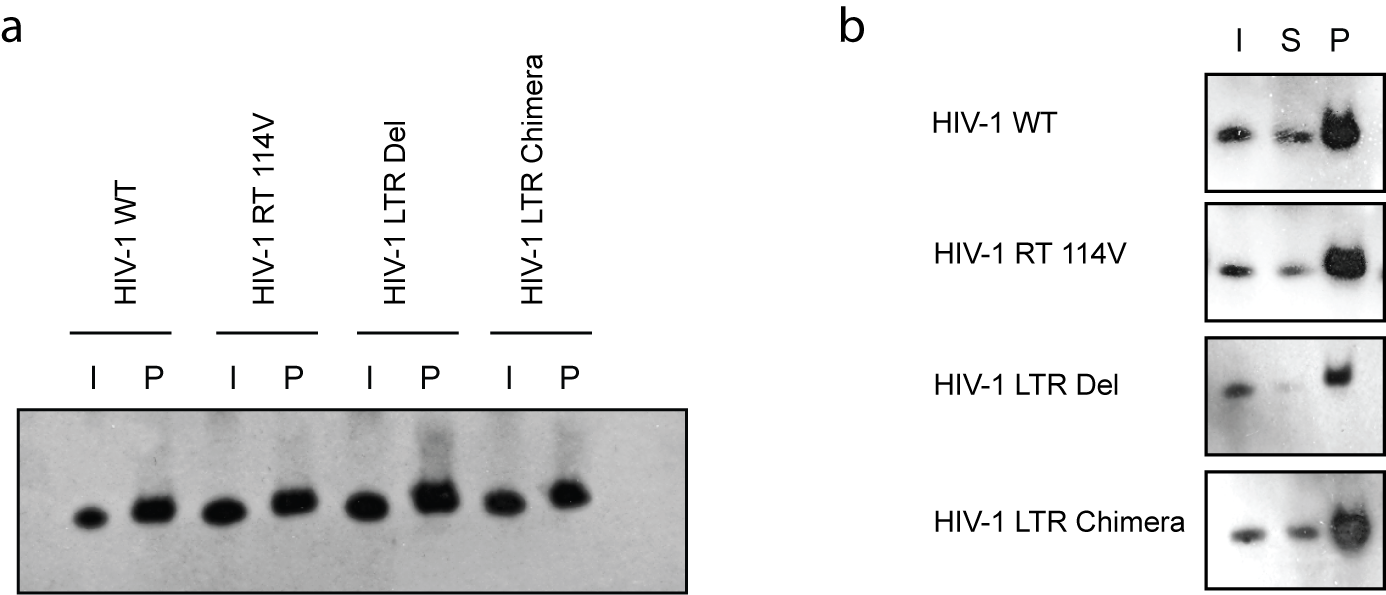

Supplement: Supplementary file 2 — 10.1186/s12977-016-0292-7 Effect of FST mutations on capsid stability. (a-b) HeLa cells were infected with WT GFP-HIV VLP, or VLP carrying LTR mutations (LTR Del or LTR Chimera). Cells were lysed (a) 2 h or (b) 20 h.p.i. and cell lysate [input, I] was separated into soluble [S] and pellet [P] fractions by centrifugation through a sucrose cushion. CA was detected by immunoblotting. Blots are representative of 3 independent experiments. [file 12977_2016_292_MOESM2_ESM.tif]
